# Crystallographic data processing for free-electron laser sources

**DOI:** 10.1107/S0907444913013620

**Published:** 2013-06-15

**Authors:** Thomas A. White, Anton Barty, Francesco Stellato, James M. Holton, Richard A. Kirian, Nadia A. Zatsepin, Henry N. Chapman

**Affiliations:** a Center for Free-Electron Laser Science, DESY, Notkestrasse 85, 22607 Hamburg, Germany; b University of Hamburg, Luruper Chaussee 149, 22761 Hamburg, Germany; c Department of Chemistry and Biochemistry, Arizona State University, Tempe, AZ 85287, USA; d Department of Physics, Arizona State University, Tempe, AZ 85287, USA; e Department of Biochemistry and Biophysics, University of California, San Francisco, CA 94158, USA; f Lawrence Berkeley National Laboratory, Berkeley, CA 94720, USA

**Keywords:** data processing, free-electron lasers, serial crystallography, *CrystFEL*, *Cheetah*

## Abstract

A processing pipeline for diffraction data acquired using the ‘serial crystallography’ methodology with a free-electron laser source is described with reference to the crystallographic analysis suite *CrystFEL* and the pre-processing program *Cheetah*. A detailed analysis of the nature and impact of indexing ambiguities is presented. Simulations of the Monte Carlo integration scheme, which accounts for the partially recorded nature of the diffraction intensities, are presented and show that the integration of partial reflections could be made to converge more quickly if the bandwidth of the X-rays were to be increased by a small amount or if a slight convergence angle were introduced into the incident beam.

## Abstract 2

A processing pipeline for diffraction data acquired using the ‘serial crystallography’ methodology with a free-electron laser source is described with reference to the crystallographic analysis suite *CrystFEL* and the pre-processing program *Cheetah*.

## 1. Introduction

A particularly successful early outcome of the availability of experimental time at the world’s first hard X-ray free-electron laser (FEL) source, the Linac Coherent Light Source or LCLS (Emma *et al.*, 2010 ▶), has been its application to macromolecular crystallography (Chapman *et al.*, 2011 ▶). While the ultimate aim of biological imaging using FEL sources is to be able to image single protein molecules (Neutze *et al.*, 2000 ▶), the study of small crystals, with dimensions ranging from a few micrometres to below one micrometre, has already shown itself to be a useful achievement in its own right. An FEL source can deliver a sufficiently large number of X-ray photons to record a diffraction pattern in a single pulse with a duration of less than 100 fs, which has been shown to be sufficiently fast to ‘outrun’ specimen damage, allowing high-resolution diffraction patterns to be recorded and structural information to be recovered even though the radiation dose is much higher than is normally tolerable (Boutet *et al.*, 2012 ▶). Because the X-ray dose to the sample is very high, the specimen will be destroyed after a single pulse, but not before the pattern has been recorded. The destruction of the crystal shortly after exposure means that a new crystal must be moved into the path of the X-rays in time for the subsequent X-ray pulse. One method by which this can be achieved is to deliver the crystals to the interaction region as a liquid suspension using a suitable injection device (DePonte *et al.*, 2008 ▶; Weierstall *et al.*, 2012 ▶). Bragg intensity measurements must be made by averaging many individual measurements because many parameters, such as the orientations of the crystals (and hence the partialities of the recorded reflections) and the intensity of the X-ray pulses, cannot easily be controlled. The methodology of recording one diffraction pattern per crystal using a free-electron laser has been given the name ‘serial femtosecond crystallography’ or SFX (Lomb *et al.*, 2011 ▶).

Diffraction patterns from an SFX experiment can be processed using modified versions of standard methods (Kirian *et al.*, 2010 ▶), the main difference being that a very large number of patterns must be processed and the resulting intensities combined in a Monte Carlo fashion. The free and open-source software package *CrystFEL* has been published in the hope that use of the technique can become as widespread as the availability of experimental facilities can permit (White *et al.*, 2012 ▶). The main aspects of serial crystallography which require modifications to the conventional analysis routes are the subject of the first five sections of this article. The final section presents some simulation results showing that the Monte Carlo integration scheme can reach convergence over the partialities more quickly if the X-ray beam were to be made slightly convergent or its spectral bandwidth were increased.

## 2. Data acquisition

**Figure 1:**
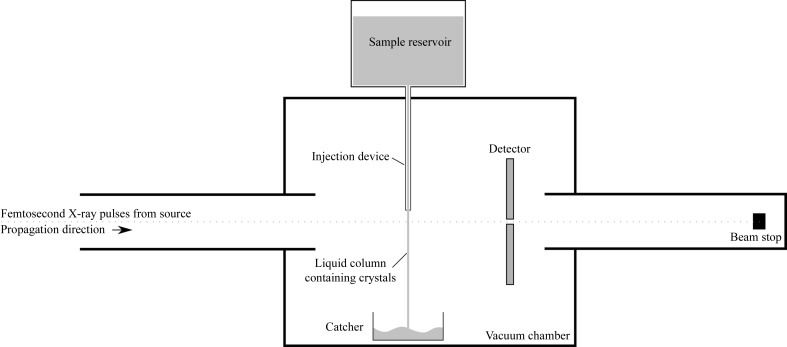
Schematic diagram of the defining features of an SFX experiment.

The defining characteristics of an SFX experiment are shown schematically in Fig. 1 ▶. Small crystals of the specimen are delivered to the beam in a liquid suspension. The liquid column is created by an injection device such as a gas dynamic virtual nozzle, which uses the focusing effect of a coaxial gas flow to create a column of liquid with a diameter of around 1 µm (DePonte *et al.*, 2008 ▶). Sample is pumped to the injection device from a reservoir, which holds sufficient sample to sustain the liquid column for the data-acquisition period (typically around 1 h at a time). Femtosecond X-ray pulses, each containing sufficient photons to allow a diffraction pattern to be recorded from a single crystal with only one pulse, are focused onto the liquid column. The diffraction patterns are recorded by a detector, which has a hole to allow the undiffracted beam to pass through, which is necessary because the undiffracted X-ray beam close to the focal point has enough power density to melt through a conventional beam stop. Beyond the interaction point, the liquid column strikes a ‘catcher’, which is a cup into which the sample residue may safely be deposited and from which it can easily be cleaned away. The injection device, liquid column, catcher and detector are all contained within a vacuum chamber. In several experiments, the injection and catcher equipment have been contained within a separate differentially pumped shroud in order to minimize the extent to which the sample can contaminate the surrounding vacuum environment (Weierstall *et al.*, 2012 ▶). A device has also been devised to avoid settling of the crystals in the sample reservoir by slowly rotating it back and forth during the experiment (Lomb *et al.*, 2012 ▶).

There are three key differences between data acquired from an SFX experiment and data acquired by the more conventional rotation method. Firstly, because the position of the crystals in the liquid column cannot be controlled, not every detector readout will contain diffraction from a single crystal. Many of the X-ray pulses will not meet a crystal and hence will produce blank detector readouts, while others may meet two or more crystals at once and produce overlapping diffraction patterns. Secondly, only one diffraction pattern can be acquired from each crystal and, because of the liquid-injection technique, each crystal will have a random orientation unless the crystals become aligned by the flow of the liquid. Thirdly, each diffraction pattern will contain partially integrated Bragg reflections because it was acquired with a single X-ray pulse and negligible rotation of the sample during the exposure. §[Sec sec3]3 describes the initial diffraction-pattern processing which addresses the first point, §§[Sec sec4] [Sec sec5] [Sec sec6]4–6 describe how the second point may be addressed and some of its consequences and finally §[Sec sec7]7 describes how the third point, that of partially integrated reflections, might be circumvented in future experiments.

## 3. Extraction and pre-processing of diffraction patterns

SFX experiments at LCLS to date have used the maximum X-­ray pulse-repetition rate of 120 Hz, yielding 432 000 detector readouts per hour and easily more than 30 million per experiment. If analysis of each frame were to take only one second, which is a conservative estimate of the amount of time required to find, index and integrate peaks and to check the results, then processing the entire experimental data set in a serial manner would take over 340 d. The European XFEL, which is scheduled to be operational from 2015, offers the potential of 27 000 pulses per second owing to its superconducting linear accelerator (Altarelli *et al.*, 2007 ▶), thus increasing data-acquisition rates even further. Efficient diffraction-pattern screening algorithms and parallelized execution are necessary to reduce the raw data stream into a more manageable set of data frames containing only diffraction patterns which have a high likelihood of being usable for auto-indexing and intensity integration. This process is implemented in the open-source program *Cheetah* and typically results in around 10% or less of the original data volume being passed on to the more time-consuming crystallographic analysis stages. *Cheetah* also applies a series of corrections to the frames, with the aim of reducing or removing electronic noise and background scatter, presenting as far as possible only the distribution of scattered intensity from the crystal for later processing. *Cheetah* was given its name to reflect the speed with which these steps must be performed in order to reduce, in a reasonable amount of time, the large number of detector readouts to a much smaller number of useful patterns.

### 3.1. Pre-processing and background subtraction

In the first stage of the processing performed by *Cheetah*, frames are corrected for static offsets measured using a series of dark frames acquired shortly before or after the acquisition of the main experimental data and without allowing X-rays into the experimental apparatus. Uncorrelated shot-to-shot fluctuations between detector segments (common mode fluctuations) and changes in offset proportional to the total signal on each detector segment (coupled drifts) are corrected. Identification of bad pixels (pixels stuck at high or low values) is performed on each frame at this stage.

After the detector corrections, background scattering is removed. The diameter of the liquid column is usually larger than the width of one of the protein crystals, resulting in diffraction patterns with a significant amount of background scattering from water compared with Bragg peak intensity. For the purposes of finding crystal ‘hits’ among blank frames, this background scattering and any residual detector offsets are removed by passing a pixel-wise median filter across the frame. In this step, the local background in the vicinity of each pixel is estimated by calculating the median value of all pixels in a box centred on that pixel. Since Bragg peaks are small in size compared with fluctuations in background signal, the median of values in this local region provides an estimate of the local background, provided there are many more background pixels in the region than peak-containing pixels. For example, for Bragg peaks averaging two pixels in diameter, a square box of five pixels per side will contain on average four peak pixels and 21 background pixels. The median of these values therefore provides a slightly biased estimate of the background level sufficient for subtraction of the local background for the purposes of peak finding. Several alternative background-subtraction schemes were attempted, such as using the blank frames surrounding the current frame to estimate background in the current frame. This works well for steady background signals and at low resolution; however, the short duration and the high coherence of X-ray pulses from the FEL produces coherent speckle patterns from the liquid column that fluctuate significantly from shot to shot. We found that the simple pixel-wise median filter described above produced frames in which the Bragg peaks were retained whilst slowly varying structure arising from the water ring and residual detector fluctuations were removed. This background-subtracted image is used for peak location, while integration of peak values as described in §[Sec sec5]5 removes any residual background and calculates the contribution to the error in the intensity measurement from the noise.

### 3.2. Assessment of diffraction quality

Bragg peaks are identified by searching for clusters of connected pixels that lie above an intensity threshold. The minimum and maximum number of connected pixels that constitute a potential Bragg peak can be specified to reject overly large or small peaks depending on the nature of the sample. This pixel-count criterion serves to reject single-pixel outliers whilst also rejecting diffuse peaks from ice nucleation in the liquid column. A constant intensity threshold performs reasonably well at low resolution, but the desire to capture weak peaks (requiring a low threshold) conflicts with the need for a higher threshold in the water-ring region where noise levels are higher. We therefore apply an additional criterion based on local noise levels, in which the peak intensity must exceed a given local signal-to-noise ratio (SNR) in order to qualify as a peak. The region used for determining signal to noise is the same as the local background region, omitting pixels within the peak itself. Only diffraction patterns with more than a certain number of peaks are retained for further processing. We found a minimum of 25 peaks to be a good compromise between retaining potentially indexable patterns and rejecting the vast majority of useless frames.

The hit rate, defined as the fraction of frames that were determined to contain diffraction patterns, is calculated as an average over blocks of 30 s (3600 frames) and written to the terminal. *Cheetah* also produces histograms showing the number of diffraction patterns with respect to both the total number of peaks and the resolution (defined as the spatial frequency corresponding to the radius of the circle, centred at the beam position, containing 80% of Bragg peaks). These statistics have provided useful fast feedback about the influence of crystallization conditions during the experiment.

### 3.3. Speed considerations

Rapid processing and evaluation of the data is critical, particularly for rapid feedback on experimental conditions during the experiment. To this end, data processing within *Cheetah* is multi-threaded. One thread reads data from disc as fast as possible, populating an event structure that is passed to a worker thread for independent processing.

Depending on the amount of computation that is required to process each image, the data-processing speed can easily become limited by the facility-provided file-reading libraries. For example, LCLS data are read using the facility-provided file-reading libraries, in which case the diffraction-pattern processing speed became limited by the code responsible for reading the data from disc. Given these limitations, a single 16-­core server becomes load limited only when performing the relatively computation-intensive task of local background subtraction.

In a typical experimental scenario, processing of diffraction patterns can be partially automated: data processing is automatically started once a run becomes available on disc, providing rapid feedback as the experiment progresses. It would be possible to pass these peak lists directly to *CrystFEL* for auto-indexing on the fly without the need to save any intermediate data to disc by taking advantage of *CrystFEL*’s shared library component libcrystfel, which allows the important routines in *CrystFEL* to be called directly from another program. Whilst this could provide some automated assessment of diffraction-pattern ‘indexability’, our experience has shown that the parameters for indexing usually require adjustment by hand. Since the number of frames of useful data is typically much smaller than the size of the raw data, it has so far been feasible to save potential crystal hits to disc for subsequent evaluation using *CrystFEL*.

## 4. Indexing and determination of the unit-cell parameters

Indexing of patterns in *CrystFEL* is performed using conventional algorithms, such as the *DPS* algorithm implemented in *MOSFLM* (Rossmann & van Beek, 1999 ▶; Powell, 1999 ▶; Powell *et al.*, 2013 ▶) or the *DirAx* algorithm (Duisenberg, 1992 ▶). The programs *MOSFLM* and *DirAx* can be applied directly to the data, one diffraction pattern at a time, using a specifically designed ‘harness’ program which supervises the execution of the program on the many thousands of patterns in an automated manner. Indexing is considered to have been performed successfully if:

(i) the lattice parameters found by the autoindexing tool match, to within a specified tolerance, the known unit cell for the structure, or can be made to match by a simple affine transformation, and
(ii) the orientation of the matching unit-cell parameters can be used to predict a reasonable number of the peaks in the diffraction pattern.

Because of the highly automated nature of the processing, indexing is less successful than would be expected in a conventional situation where human intervention would be applied in difficult cases. The success of the indexing process is therefore quantified as the ‘indexing yield’, which is defined as the fraction of the input patterns that could be successfully indexed. The indexing yields in experiments so far have varied widely, with maximum values of around 40%. The indexing yield was typically lower when the unit-cell parameters were very large (giving spots with very small separations on the detector), when the diffraction patterns did not contain spots at high angles, when the camera length (the distance from the sample to the detector) was too short, resulting in the spots being confined to the middle of the detector instead of being spread over its entire surface, or when the input patterns were ‘contaminated’ significantly with images falsely identified as hits by imperfections in the pre-processing stages.

The above description requires ‘known’ lattice parameters against which to compare the cells from the autoindexing tool. In many cases so far this has been obtained from the same (or similar) crystals analyzed at a synchrotron facility. In other cases, it has been possible to determine the correct lattice parameters by skipping the cell comparison [see (i) above] and plotting histograms of the resulting lattice parameters. This procedure relies on the ability of the autoindexing tool to produce consistent lattice parameters when presented with patterns from different orientations of the same crystal structure. Not only must the correct lattice parameters be found each time, but the correct representation of the unit cell must be chosen from the many possible unit-cell choices which represent the same lattice. It was found that both *DirAx* and *MOSFLM* were able to provide this, and in most cases so far it has been found that the correct lattice parameters emerged from such a procedure. Furthermore, the sharpness of the peaks in the histograms can be used as a measure of the accuracy of experimental parameters such as the detector layout and the position of the central beam on the detector. Once the lattice parameters have been determined, the indexing process can be repeated including the cell-comparison step (i).

It is clear that the procedure described above, in which the lattice parameters of each individual diffraction pattern are determined *ab initio* and compared against reference parameters, is not optimal. In reality, it can be assumed that the true parameters are known and a search can be performed specifically for lattice vectors which have moduli and angular relationships similar to the known values. An experimental implementation of such a procedure is included in *CrystFEL* and has been given the name *ReAx*. The method proceeds in a manner similar to the fast Fourier transform (FFT) autoindexing algorithm described by Steller *et al.* (1997 ▶), as follows.

Firstly, the three-dimensional positions of the peaks in the diffraction pattern are calculated by mapping the peaks onto the Ewald sphere in reciprocal space. As articulated by Leslie (2006 ▶), an uncertainty exists in the three-dimensional position if the volume of reciprocal space which can contribute to the pattern is significant; for example, if the crystal is rotated by a large amount during the X-ray exposure. In the ‘snapshot’ regime which results from the short duration of the X-ray pulses from an FEL source, the crystal motion is negligible. Nevertheless, a significant volume may still result if the spectral bandwidth or the convergence angle of the X-ray beam is large enough. It is assumed that the true three-dimensional location of the corresponding reciprocal-lattice point is given by the nominal wavelength of the X-ray beam and that the convergence angle of the beam and the mosaicity of the crystal are both exactly zero. Under this assumption, the peak locations in each diffraction pattern can be mapped into exact locations in reciprocal space. If the bandwidth, convergence angle or mosaicity values are large enough to introduce a significant error into the locations of the reciprocal-lattice points then the success rate of indexing is expected to be correspondingly lower (Powell *et al.*, 2013 ▶).

Once three-dimensional positions have been determined for all of the peaks, an FFT vector search similar to that described by Steller *et al.* (1997 ▶) is performed. However, instead of searching for the most dominant interplanar vectors in reciprocal space, it searches for strong candidate vectors with lengths within a certain tolerance (10% in the initial version) of the reference parameters. Once the search has been completed, the candidate vectors are ‘squashed’ as described by Steller *et al.* (1997 ▶), discarding candidates that are close to other candidates with higher figures of merit. After the ‘squashing’ procedure has been completed, the remaining vectors are refined using an iterative form of the single-step refinement procedure given by Clegg (1984 ▶). The iteration is required because the implementation includes peaks in the refinement calculation only if they are already sufficiently close to lattice points of the candidate vector, in an attempt to reduce the impact of spurious peaks in the diffraction pattern. Once the final list of candidates has been created, the program attempts to assemble the vectors into a unit cell with interaxial angles which match the reference cell. The FFT vector search cannot distinguish between the vectors **v** and −**v**, and thus the cell-assembly routine must try both possibilities for each candidate. Only a right-handed combination of vectors will be accepted by the program. The indexing solution is chosen in which the highest number of peaks in the pattern are close to reciprocal-lattice points.

## 5. Integration and merging of the intensities

After each pattern has been indexed, the peak intensities must be measured and the errors in the measurements estimated. The combined process of integration and merging, as implemented in *CrystFEL*, differs slightly from the previously described method (Kirian *et al.*, 2010 ▶). In the earlier method, the diffraction geometry produced by the autoindexing procedure was used to determine which pixels in the frame were within a certain fixed distance of the nearest reciprocal-lattice point. In the new method, peak positions are predicted by a geometrical calculation, integration is performed at each predicted location and the mean of all intensity observations for each symmetrically unique reflection is then taken. In the previous method, the integrated intensity was taken to be the pixel-wise averaged intensity corrected for the solid angle of each pixel viewed from the interaction region. In the new method, the integrated intensity is taken to be the average total peak intensity, which does not require solid-angle corrections. The new method is much less sensitive to the size of the integration domain.

**Figure 2:**
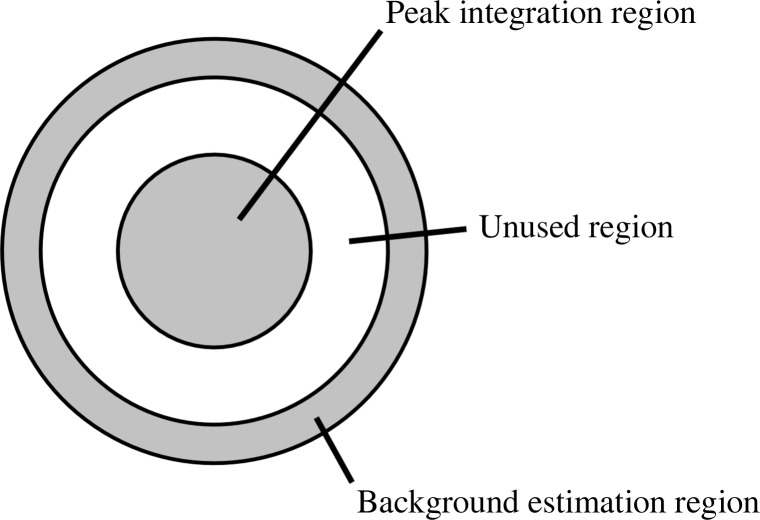
Schematic diagram of the peak-integration scheme used by *CrystFEL*.

As of v.0.3.0, *CrystFEL* uses a simple summation integration method based on three concentric circles around the predicted location of a spot, similar to the method used by *DENZO* except without profile fitting (Otwinowski & Minor, 1997 ▶). A schematic diagram is shown in Fig. 2 ▶. The radii of the circles must be configured specifically for each experiment, because the sizes of the spots and the distances between them depend on characteristics of the crystals, the detector geometry, the X-­ray parameters and the lattice parameters. Typical values have been around three, four and five pixels for the three radii. The peak-integration region should be sufficiently large as to cover the entire area of each peak, allowing a small amount of extra space to allow for inaccurately calculated spot positions. The background-estimation region should be as large as possible without causing it to contain other reflections. If the background-estimation region is too small then the estimation of the background level and hence the measurement of the peak intensity will be inaccurate. To avoid problems with closely spaced reflections, pixels in the background-estimation region are ignored if they are also within the peak-integration region of any other reflection. The intensity of the peak is measured using the pixels within the innermost circle and the background is characterized from the pixels between the outer and middle circles, leaving an unused annular region between the two. The unused region acts as a buffer to avoid wildly overestimating the background in the event that the predicted location of the peak is wrong by a small amount, which would otherwise place the peak in the background region. The mean of the pixel intensities in the outer annulus is subtracted from that of each pixel in the inner region, and the resulting values are summed. Intensities and their standard errors are estimated in the same units as the pixel values are represented in the image data. The standard error in the intensity of the peak is estimated by combining contributions from the variance of the background over the peak region as well as counting statistics applied to the number of photons in the peak, 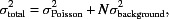
where σ^2^_background_ represents the variance of the pixel intensities in the background region, *N* is the number of pixels comprising the peak-integration area (the central shaded region in Fig. 2 ▶) and σ^2^_Poisson_ = 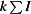. Here, 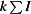 represents the total intensity in the peak region measured in detector intensity units after subtraction of the background and *k* is the number of detector intensity units arising from one photon at the incident beam energy.

Since the X-ray beams from a free-electron laser source are highly coherent, if the crystals are very small then Fourier truncation may dominate the size of the intensity distribution surrounding each reciprocal-lattice point. If the resulting truncation fringes can be resolved sufficiently well then they can be used to reconstruct an image of the crystal itself (Chapman *et al.*, 2011 ▶), and it has been proposed that they might provide a new route to experimentally measured phases (Spence *et al.*, 2011 ▶). However, they present a nuisance in the context of traditional crystallography because they lead to large differences between the shapes of Bragg peaks within an individual diffraction pattern, as shown in our previous article (White *et al.*, 2012 ▶), defeating conventional two-dimensional profile fitting. A better strategy for dealing with this situation would be to use three-dimensional profile fitting on a shot-by-shot basis by making use of the fact that a truncated direct-space lattice gives rise to translational symmetry in reciprocal space and thus (in the absence of other effects) the profile should be the same for all reciprocal-lattice points. A model of the underlying profile shape in reciprocal space, as well as the beam conditions, could then be optimized for best fit with the observed two-dimensional peak profiles in each diffraction pattern. Such a method would have the added advantage of producing information about the crystal size and shape, which could be correlated with the strength of diffraction in each pattern and the information used to help optimize the crystallization conditions. However, considerable further development will be necessary before such a method becomes a reality.

## 6. Indexing ambiguities

In many crystal structures a transformation exists which overlays the lattice with itself but does not superimpose the actual structure on itself in the same orientation. In these cases, the transformation is part of the symmetry of the lattice but not of the structure itself. The transformation is often a rotation of 180° about a certain direction. Since conventional indexing algorithms operate on a geometrical description of the lattice only, they are unable to distinguish between these two (or sometimes more) orientations and there is an equal probability that a pattern would be assigned either of them. There is therefore an ambiguity of orientation remaining after the indexing procedure. When the ‘serial’ approach is used, acquiring only one pattern from each one of many crystals in unrelated orientations, each diffraction pattern must be indexed individually and the indexing will be inconsistent with roughly equal fractions of the patterns falling into each of the possible orientations. Because the structure appears differently in the two orientations, this would result in reflections being confused with otherwise unrelated reflections and their intensities being incorrectly mixed together. The end result after merging many diffraction patterns would be as if the intensities had been collected from perfectly twinned crystals, even if the real crystals were not physically twinned.

The overlap of the lattice with itself can be exact or can be approximate. Exact overlaps occur for chiral crystals if the point group is 3, 312, 321, 4, 6 or 23, but not for point groups 1, 2, 222, 422, 622, 432 or 32. Approximate overlaps cause indexing ambiguities when a transformation causes the lattice to overlap with itself to within the indexing tolerance mentioned in §[Sec sec4]4 and can exist in addition to any exact overlaps which may occur. Further possibilities exist for achiral crystals, but these are not normally relevant to biological crystallo­graphy and will not be discussed here.

The transformations corresponding to any indexing ambiguity can be identified by left coset decomposition of the lattice point group with respect to the true point group (Flack, 1987 ▶). This process can be thought of as ‘division’ of point groups. Given a high-symmetry point group (such as that of the lattice) and a low-symmetry point group (such as that of the crystal structure), left coset decomposition yields the symmetry operations which must be added to the low-symmetry point group to yield the high-symmetry point group, taking into account that individual symmetry operators must themselves appear multiple times as dictated by all of the other operators. The resulting symmetry operator defines the indexing ambiguity. If more than one ambiguity exists at the same time, the process will yield multiple symmetry operators. If the true point group of the crystal structure is known, left coset decomposition will immediately list the indexing ambiguities. If the true point group is unknown, left coset decomposition could be performed in turn using all of the possible point groups which are compatible with the lattice, yielding a list of ambiguities for each possibility. The ambiguities could then be given (as ‘twin operators’) to the software used in later stages of analysis.

The possibilities for indexing ambiguities are very closely related to the possibilities for crystal twinning, and the literature contains lists of permitted twinning operators (Le Page *et al.*, 1984 ▶). The transformations corresponding to ambiguities can be obtained from the same tables provided that some operators are disregarded: there is no possibility that the right-handed set of lattice basis vectors could be mistaken for an alternative left-handed one by the indexing algorithm, and therefore indexing ambiguities cannot add a mirror plane or inversion centre to a chiral structure. Usefully, this means that indexing ambiguities cannot lead to each reflection being confused with its Friedel opposite and hence to the destruction of an anomalous signal. However, since each one of the reflections in a Bijvoet pair is equally likely to be the strongest, merging of unrelated reflections will result in the anomalous signal being reduced by a factor of 2^1/2^, as well as causing further problems later on while solving the structure by this method.

In conventional data acquisition and processing, the crystallographer must be aware of indexing ambiguities whenever data sets from more than one crystal are indexed independently and then merged or compared, and in such cases they must ensure that the reflections were indexed consistently between all crystals. Examples of such situations would be when combining partial sweeps of reciprocal space from multiple crystals or when comparing intensities from a native protein with a heavy-atom derivative to perform SIR. Ambiguities can be resolved in conventional data processing by comparing the fit of one data set to another in each of the possible orientations and accepting the orientation with the closest match of the intensities. However, all attempts so far to apply such a process to SFX data have not met with success. The true intensities of the reflections are obscured behind a large amount of noise, which is not currently understood, and therefore the correlation procedure does not clearly reveal the correct orientation. The same sources of error currently mean that a very large number of intensity measurements must be combined to obtain accurate merged intensities. Previous articles have attributed this noise to incomplete integration of the reflection, which cannot be accurately compensated for in the current version of *CrystFEL*. Until the main sources of error in SFX data are understood and compensated for sufficiently accurately to reveal the true orientation of the crystal in a single pattern, data measured from merohedral or pseudo-merohedral crystals will suffer from indexing ambiguities. For the time being, the most appropriate course of action is to merge the reflections according to the apparent symmetry rather than the true symmetry, so that all of the available measurements are combined to provide the best quality merged intensities, albeit those apparently from a twinned crystal. This is the standard procedure when using *CrystFEL*, and a set of tables distributed with the software allows the appropriate point group to be determined at a glance. A compact version of this is shown in Table 1 ▶. For each true point group, the table shows the apparent point group after the exact indexing ambiguities have been taken into account. For example, a structure with space group *P*3_1_21 has point group 321, which according to the table must be merged according to point group 622. Approximate indexing ambiguities, which depend on the lattice parameters as well as the point group, are not shown in the table and must be accounted for separately.

The implications of indexing ambiguities for future structure-solution attempts using data treated using the Monte Carlo technique must be seen in perspective. The possibility must always be considered, for any crystallographic data, that the crystal was physically twinned. The crystallographer must consider in all cases that the true symmetry may be a subgroup of the apparent symmetry. The relationships between the apparent symmetry and possible true symmetries are clearly defined, and the possibilities are usually not so numerous that they cannot all be tried in turn to find the correct answer.

Future possibilities for the resolution of indexing ambiguities in serial femtosecond crystallography might include comparing intensities against a merged data set, perhaps using a probabilistic method in which the need to make a ‘hard’ assignment of orientation is avoided until a sufficient number of patterns have been resolved so as to make the solution clear, in the spirit of the *EMC* algorithm (Loh & Elser, 2009 ▶). Another possibility might be to alternate rounds of ambiguity resolution with rounds of model fitting by post-refinement (Rossmann & van Beek, 1999 ▶). With the difficulties which arise from twinning in the structure-solution process, a great deal of effort is likely to be expended in the near future in this direction.

## 7. ‘Monte Carlo’ simulations

The ‘Monte Carlo’ method for merging reflection intensity measurements consists of simply taking the mean of a large number of individual measurements of the intensity of each symmetrically unique reflection from different crystallites. The method aims to provide angular integration in the absence of a goniometer, since it is obviously impossible to rotate the crystal by more than a negligible amount during the few tens of femtoseconds during which X-ray exposure occurs. Nevertheless, there are some FEL beam properties that may be tuned to produce a similar effect. One such parameter is the X-ray spectral bandwidth, and another is the convergence angle of the beam as it strikes the crystal. The bandwidth can be altered by altering the characteristics of the electron beam in the accelerator and the convergence angle of the X-ray beam is determined by the focusing mirrors or lenses upstream of the interaction region. Previous literature has investigated the convergence of Monte Carlo merging with respect to the integration-domain size, which is the maximum allowable distance between the calculated location of a pixel in reciprocal space and a reciprocal-lattice point (Kirian *et al.*, 2010 ▶). The previous analysis also used simulated diffraction patterns and included the autoindexing procedure. However, a partiality calculation can be used to calculate the intensities of partial reflections and hence evaluate the contribution to the convergence of the Monte Carlo procedure which arises purely from the partialities of the reflections. This section describes a set of simulations performed in this manner, with the aim of determining how strongly the beam characteristics can affect the quality of the data.

**Figure 3:**
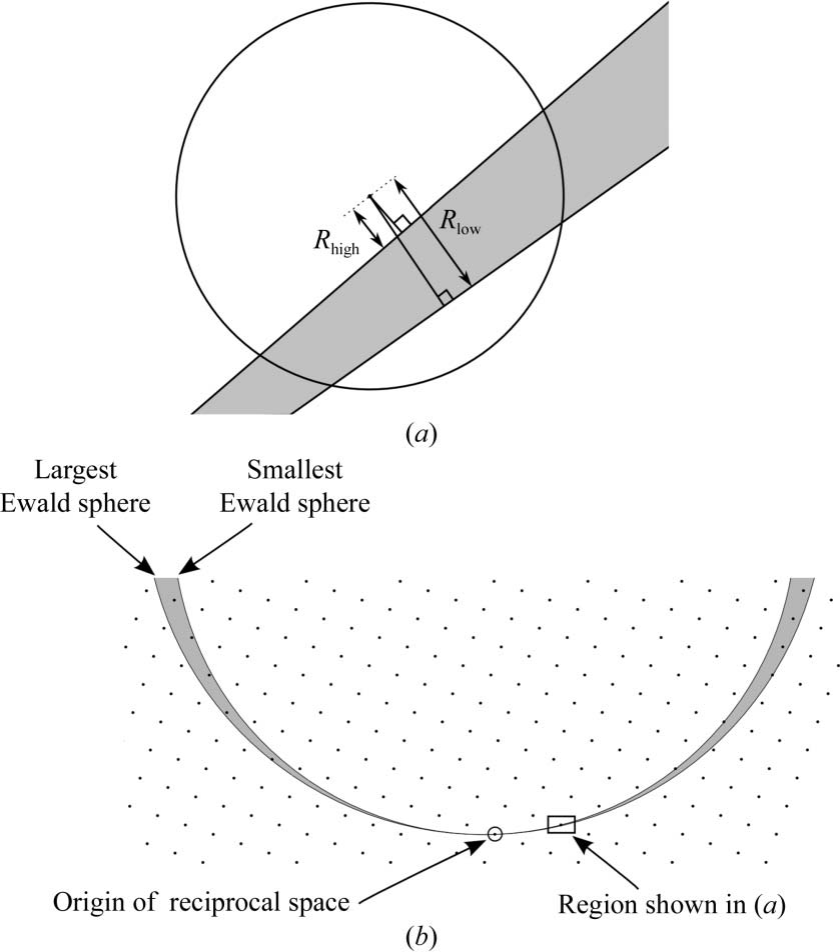
(*a*) Geometrical model used for the calculation of spot partialities. (*b*) Context of the diagram.

Fig. 3 ▶ shows a simple reflection profile approximated by a sphere, of which a central section is shown in the plane containing both the incident and scattered wavevectors. The shaded area represents the volume of the reciprocal space in the vicinity of the reciprocal-lattice point which could potentially be excited under a particular set of beam conditions. The upper extent of the shaded area corresponds to the smallest Ewald sphere radius, which itself corresponds to the longest wavelength in the spectrum of the incident X-ray beam. The lower extent of the shaded area corresponds to the largest Ewald sphere radius and the shortest wavelength in the spectrum of the incident X-ray beam. In the simulations presented here, the spectrum of the X-ray beam was assumed to be a flat rectangular distribution in this simplified model. The upper and lower surfaces are not exactly flat nor parallel, but the deviation is small because the size of the profile is very small compared with the radius of the Ewald sphere. For each reciprocal-lattice point which fell within a sphere with a radius equal to the resolution limit of the detector, the distances *r*_low_ and *r*_high_ were calculated by considering limiting Ewald spheres at the two extremes of the X-ray spectrum. To calculate the effect of a convergent X-ray beam, the position of the centre of the Ewald sphere was rotated about the origin of reciprocal space in a direction away or towards the reciprocal-lattice point under consideration by an angle of half the specified beam convergence. The distance *r*_high_ corresponds to the limiting Ewald sphere corresponding to the longest wavelength and tilting away from the reciprocal-lattice point, whereas *r*_low_ corresponds to the shortest wavelength and tilting towards the reciprocal-lattice point. Positive values of *r*_low_ or *r*_high_ correspond to the reciprocal-lattice point being inside the Ewald sphere, consistent with the definition of excitation error used in electron microscopy (Spence, 2003 ▶). From the values of *r*_low_ or *r*_high_ and the profile radius, the partiality of the reflection was calculated using the expression given by Rossmann *et al.* (1979 ▶). The partial intensity for the reflection was calculated as the fully integrated intensity (from a prior structure-factor calculation) multiplied by the partiality, and then multiplied by a ‘Lorentz factor’ proportional to *r*_low_ − *r*_high_, which models that the radiation is more ‘spread out’ at high scattering angles where the smallest and largest Ewald spheres separate further than at lower angles. If any part of the profile was determined to be within the excited volume of reciprocal space, the location of the peak in the diffraction pattern was calculated. If the calculated location of the peak fell within the active region of the detector, the partial intensity for that peak was written to disc.

The program *partial_sim* from *CrystFEL* was used to repeat this process for a large number of random orientations and the partial intensities were combined to produce the final intensities using the Monte Carlo merging program *process_hkl*. As a test sample, we used proteinase K (PDB entry 2prk; Betzel *et al.*, 1988 ▶), which crystallizes in space group *P*4_3_2_1_2 with unit-cell parameters *a* = 68.17, *c* = 108.26 Å. The photon energy at the centre of the spectrum was 900 eV. The simulated detector was square, with a side length of 76.8 mm at a distance from the interaction region of 70 mm, giving a resolution of 2.8 Å at the edge and 2.1 Å at the corner. The radius of the profile was 3 × 10^−4^ Å^−1^. Overall scaling factors were generated for each pattern randomly with a Gaussian distribution of standard deviation 0.3 centred on unity. Fully integrated intensities were calculated using *ano_sfall.com* (http://bl831.als.lbl.gov/~jamesh/mlfsom/ano_sfall.com). Gaussian noise with a constant standard deviation of 10 was added to all of the intensities, which corresponded to approximately 10% of the mean fully integrated intensity at the corner of the simulated detector or 2% of the mean intensity of the lowest order reflections. We then calculated the effect of the bandwidth and the beam-convergence angle on *R*_split_, which measures the agreement between the sets of intensities created by merging odd- and even-numbered patterns from the overall data set^1^,
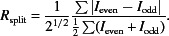
Two sets of simulations were performed to investigate the effect of the bandwidth and the beam-convergence angle on the convergence of *R*_split_. In the first set of simulations the beam-convergence angle was fixed while the bandwidth was varied, and in the second set the bandwidth was fixed while the beam-convergence angle was varied. In both cases, the value of the fixed parameter was set at a realistic value from experiments at LCLS: 1 mrad for the beam-convergence angle and 0.1% for the bandwidth.

**Figure 4:**
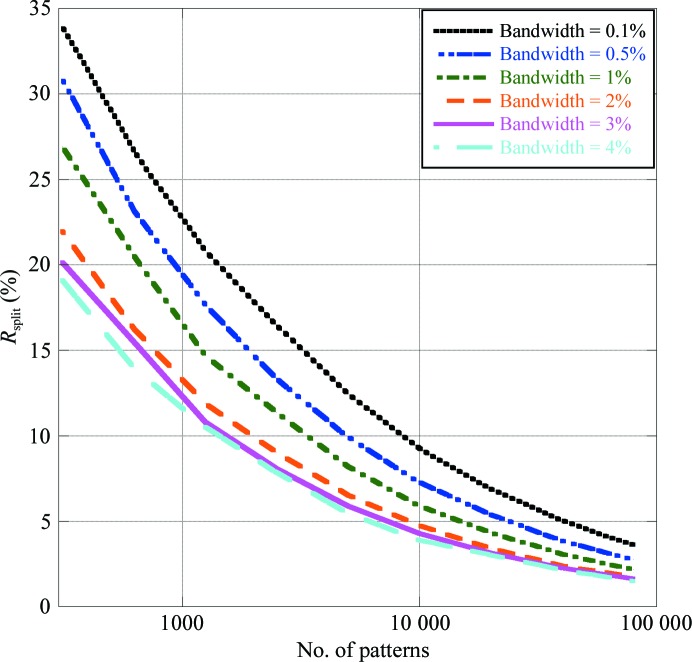
The effect of spectral bandwidth on Monte Carlo integration.

Firstly, simulations were performed with the beam-convergence angle fixed while varying the bandwidth from 0.1 to 4%. The justification for these choices was that 0.1% is approximately the estimated bandwidth at which LCLS is currently operational, while 4% is the limit that new machines currently being designed will reach. In particular, SwissFEL will be able to provide at least 1.2% bandwidth pulses (most likely up to 3.5% and perhaps higher) when operated in ‘energy chirp mode’ (Patterson *et al.*, 2010 ▶). Fig. 4 ▶ shows the behaviour of *R*_split_ as a function of the number of patterns in the overall data set for the different bandwidth values. It is evident that the higher the bandwidth is, the fewer patterns are needed to achieve a given *R*_split_. For example, *R*_split_ = 10% for 8000 patterns using a 0.1% bandwidth beam, but for about 1500 patterns using a 4% bandwidth beam. To achieve *R*_split_ = 5% about 40 000 patterns are needed with 0.1% bandwidth, while only about 6000 are necessary with 4% bandwidth.

**Figure 5:**
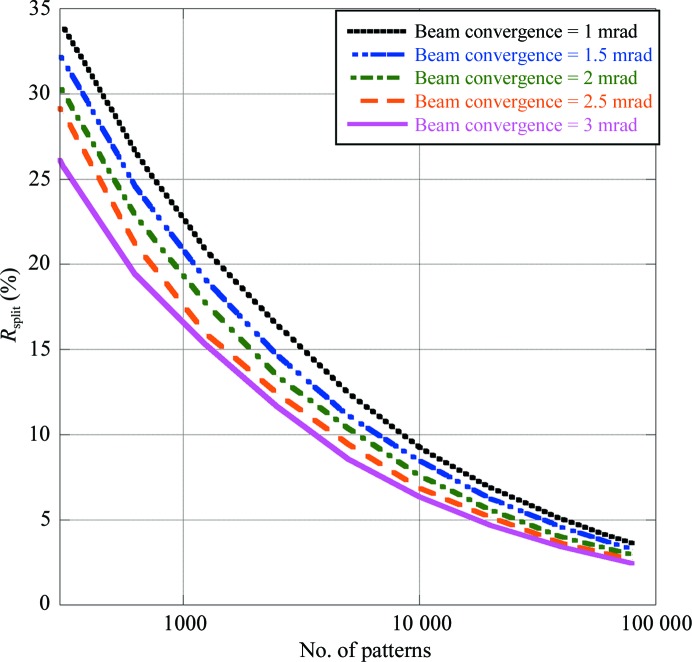
The effect of the beam-convergence angle on Monte Carlo integration.

Secondly, the beam-convergence angle was varied from 1 to 3 mrad while keeping the bandwidth fixed at 0.1%. The results are shown in Fig. 5 ▶ as a function of the number of patterns. Again, with a higher beam-convergence angle fewer patterns are needed to achieve a particular value of *R*_split_. For example, *R*_split_ = 10% is obtained with 8000 patterns using a beam with 1 mrad convergence, but with less than 4000 patterns using a beam with 3 mrad convergence. To achieve *R*_split_ = 5% about 40 000 patterns are needed for 1 mrad, whereas fewer than 20 000 patterns would be needed with a convergence angle of 3 mrad.

The values obtained for *R*_split_ in this simulation are low compared with those encountered in experiments to date, and this suggests that factors other than the reflection partialities may dominate the convergence of the Monte Carlo process in a real experiment. Possible factors include detector readout noise and nonlinearity, Poisson noise at low signal levels and a wider distribution of scaling factors or an X-ray spectrum that differs from the rectangular approximation used here. Spreading the available intensity over a wider bandwidth or convergence angle would lead to a reduction in the overall intensity of the diffraction pattern and therefore make some of these problems worse, and no attempt has been made here to determine whether or not these sources of error do in fact dominate in serial crystallography experiments at free-electron laser sources. In addition, increasing these parameters to large values may cause problems with indexing, as discussed in §[Sec sec4]4. Further simulations of the type described in this section, isolating individual contributions to the error and determining their dependence on the experimental conditions, must be performed with the ultimate aim of increasing the overall data quality to make the best use of the small amount of measurement time currently available for such experiments.

## 8. Conclusions

This article has described aspects of crystallographic data processing which are relevant to serial crystallography using free-electron laser sources. However, as synchrotron-based macromolecular crystallography experiments increasingly tend towards smaller and smaller crystals it is possible that some of the concepts described in this article will find use outside FEL experiments. A possible situation might be one in which very small crystals require very high X-ray flux densities to produce measurable signals, yet the crystal is so sensitive to radiation that it can yield only a single diffraction pattern before the crystal becomes unusably damaged and a new one must be used instead. In view of the great progress made in crystallographic data analysis since the very first macromolecular diffraction studies, it seems likely that improvements will be made in all aspects of the necessary analysis in the near future.

## Figures and Tables

**Table 1:** The apparent symmetry, after taking indexing ambiguities into account, for all chiral point groups Rows with no entry under ‘Apparent point group’ exhibit no indexing ambiguity unless the lattice ‘accidentally’ appears to have a higher symmetry, as discussed in the main text.

Lattice type	True point group	Apparent point group
Triclinic	1	
Monoclinic	2	
Orthorhombic	222	
Tetragonal	4	422
Tetragonal	422	
Rhombohedral	3	32
Rhombohedral	32	
Hexagonal	3	622
Hexagonal	6	622
Hexagonal	312	622
Hexagonal	321	622
Hexagonal	622	
Cubic	23	432
Cubic	432	

## Footnotes

1 For convenience in this simulation, the two sets of intensities were created by merging patterns from the first and second halves of the overall data set. Since the relevant parameters were randomly generated for each simulated pattern, this should not affect the results. However, it would be important to interleave the selection of patterns when using real experimental data because the values of the relevant parameters may drift over the course of the data acquisition.
